# The healthcare experiences of rural-living Canadians with and without a primary care provider: a qualitative analysis of open-ended cross-sectional survey responses

**DOI:** 10.1017/S1463423624000677

**Published:** 2025-01-06

**Authors:** Kathy L. Rush, Cherisse L. Seaton, Lindsay Burton, Mindy A. Smith, Eric P.H. Li

**Affiliations:** 1 School of Nursing, University of British Columbia, Okanagan Campus, Kelowna BC V1V 1V7, Canada; 2 Patient Partner, Patient Voices Network, British Columbia, Canada and Department of Family Medicine, Michigan State University, East Lansing, Michigan, United States; 3 Associate Professor and Principal’s Research Chair (Tier 2) in Social Innovation for Health Equity and Food Security, Faculty of Management, University of British Columbia, Okanagan Campus, Kelowna BC V1V 1V7, Canada

**Keywords:** unattached patients, primary care, rural, provider shortages, experience, qualitative

## Abstract

**Aim:**

This study aimed to explore healthcare experiences of rural-living patients both with (attached) and without (unattached) a local primary care provider.

**Background:**

Primary care providers serve a gatekeeping role in the Canadian healthcare system as the first contact for receiving many health services. With the shortage of primary care providers, especially in rural areas, there is a need to explore attached and unattached patient experiences when accessing healthcare.

**Methods:**

A cross-sectional survey of rural patients both with (attached) and without (unattached) a primary care provider was conducted July–September 2022. An open-ended question gathered participants’ thoughts and experiences with provider shortages.

**Findings:**

Overall, 523 (Mean age = 51 years, 75% female) rural British Columbia community members (306 attached; 217 unattached) completed the survey. Despite similar overall health, unattached patients received care less frequently overall compared to attached patients, including less frequent non-urgent and preventive care. The vast majority of attached patients sought care from a regular provider whereas unattached patients were more likely to use walk-in, emergency department, and urgent care and 29% did not seek care at all. Overall, 460 (88.0%) provided a response to the open-ended doctor shortage question. Similar themes were found among both attached and unattached participants and included: i) the ubiquity of the doctor shortage, ii) the precariousness or fluidity of attachment status, and iii) solutions and recommendations. Greater attention is needed on the negative and cyclical impacts provider shortages have for both attached and unattached patients alike.

## Background

Family physicians serve as a central hub in the Canadian health care system, managing health and illness for patients, families, and communities. They provide direct care and make referrals to preventive services and specialist care. Evidence indicates that patients who have comprehensive and continuous family physician care over time experience positive health outcomes, including reduced all-cause and disease-specific mortality (Gray *et al.*, 2018; Kolber *et al.*, 2023). Moreover, such care leads to reduced costs by decreasing emergency department (ED) use, after-hour care, and hospitalizations (Sandvik *et al.*, 2022; Kolber *et al.*, 2023). Canadian studies show that in rural communities, retention of family physicians decreases hospitalization rates by 6–20% (Knight *et al.*, 2017; Mathews *et al.*, 2023).

However, there is a severe shortage of family physicians and nurse practitioners in Canada, resulting in approximately 6.5 million Canadians, roughly one in five, without access to primary medical care. This represents an increase of 2 million since 2019 (Duong and Vogel, 2023), with a shortfall of about 44 000 physicians predicted by 2028, with primary physicians accounting for 72% of the deficit (Richardson and Hussain, 2022). British Columbia has one of the highest Canadian family physician shortages at 27% and is among the provinces with the fewest family physicians per 100 000 in the population (Duong and Vogel, 2023; Li *et al.*, 2023). This unprecedented shortage is driven by lack of data for human health resource (HHR) planning, an aging workforce and retirements, changing expectations of family physicians, limited support and resources, outdated physician compensation, and high clinic operating costs (Li *et al.*, 2023).

Rural patients bear the greatest burden from the doctor shortage for many reasons (Fleming and Sinnot, 2018). Although approximately 18% of Canadians live in rural communities, only 8% of physicians practice there (Canadian Institute for Health Information, 2022) and those who do retire earlier (Hedden *et al.*, 2017) and are challenged to find replacements for their practices (Silver, 2017). British Columbia (BC) rural family physician turnover and retention has contributed further to the shortage; although the extent of physicians leaving practices is unknown, government investments reflect the severity of the problem (The Canadian Press, 2024). In addition, rural citizens are older, have more chronic conditions and fewer healthcare resource options than their urban counterparts (Morra *et al.*, 2011; Sibley and Weiner, 2011). News outlet data on rural ED closures due to staff shortages and residents indicate loss of more than 120 days of access in 2022, with patients diverted distances of nearly 200 kms (Kulkarni, 2022), and some closures resulting in patient deaths (Gamage, 2023). Owing to provider and other healthcare-related shortcomings, rural residents have 2 to 3 times longer travel distance to medical care compared to their urban counterparts, leading to fewer visits for routine follow-up care (Arcury *et al.*, 2005; Krasniuk and Crizzle, 2023).

Various solutions are being considered for addressing doctor shortages in Canada and patients are important partners in these efforts (Government of Canada, 2023b). However, there is little research on understanding rural residents’ perspectives on the physician shortage. Mui *et al.* (Mui *et al.*, 2020) found that rural Virginia residents who lost their local physician experienced access to care concerns and challenges and a change in their former relationally-based, person-centered care. Marshall *et al.*’s (2022) realist-informed qualitative study of nine unattached (not specifically rural) patients from Nova Scotia, Canada, found patients ‘giving up’ on finding a healthcare provider, not seeking care, and experiencing distress and concern for the future (Marshall *et al.*, 2022). We found no studies that addressed the thoughts and experiences of rural citizens around doctor shortages, whether attached or unattached to a primary care clinician. Therefore, the purpose of this study was to explore the healthcare experiences of attached and unattached rural patients.

## Methods

### Design

A cross-sectional survey design was used combining both closed and open-ended questions to gain a comprehensive and in-depth understanding of the healthcare experiences among rural attached and unattached patients. Our definition of rural was based on a community and hospital classification that categorized communities with limited general inpatient care and populations under 20 000 as rural (BC Ministry of Health, 2015).

### Sample and recruitment

Data were collected using an online cross-sectional survey of rural BC patients both attached and unattached to a primary care provider. Participants completed an eligibility question that asked ‘Are you living somewhere in British Columbia that would be considered rural or remote (e.g., population less than 20 000)?’ and those who selected ‘no’ exited the survey. The survey was promoted via rural community social media pages, rural newsletters, REACH BC, and the Patient Voices Network to recruit the convenience sample. Participants could choose to be entered into a draw for one of three gift certificates valued at CAD50.00.

### Data collection

Participants completed a 10-minute online Qualtrics questionnaire hosted by the lead author’s home institute. The investigator generated survey included six demographics questions, and nine questions based on a previous study of unattached patients (Rush *et al.*, 2022) each consisting of several response options with instructions to ‘select all that apply’ (described below).

#### Social demographics

Questions encompassed age, sex, marital status, ethnicity, employment, and household income.

#### Attachment

Questions included whether respondents had a *local* regular primary care provider (family doctor or nurse practitioner) and length of attachment or unattachment. Patients who did not have a local provider (defined as unattached) were asked whether they had a provider elsewhere they could travel to for care (and what distance).

#### Healthcare usage

Healthcare usage questions included 3 questions. First, participants were asked where they sought routine care and asked to select all that apply from a checklist of 8 options (see Table 1), and they could select ‘other’ and specify alternate sources. Second, participants were asked how often they sought care in the previous year (response choices: 0 times, 1-2, 3-4, and 4+ times). Third, participants were asked to select all the kinds of health care they sought in the past year (non-urgent, urgent, and screening/preventive care), and participants could select ‘other’ to describe the care they sought.

**Table 1.** Health characteristics and healthcare usage

Characteristic	Overall, N = 523^†^	Attached, N = 306^†^	Unattached, N = 217^†^	P-value^‡^
Time with (attached) or without (unattached) a HCP				
1-6 months	NA	28 (9.2%)	37 (17.2%)	NA
Over 6 months to 2 years	NA	56 (18.4%)	55 (25.6%)	
Over 2 years to 5 years	NA	83 (27.3%)	60 (27.9%)	
Over 5 years to 10 years	NA	75 (24.7%)	32 (14.9%)	
Over 10 years	NA	62 (20.4%)	31 (14.4%)	
Do you have a provider you can still travel to for care?				NA
Yes	NA	NA	37 (17.3%)	
No	NA	NA	177 (82.7%)	
When you need healthcare, where do you seek it? (select all that apply)?^§^				
Regular provider	283 (54.1%)	261 (85.3%)	22 (10.1%)	< .001
Walk-in or healthcare clinic	157 (30.0%)	54 (17.6%)	103 (47.5%)	< .001
Emergency department (routine care)	73 (14.0%)	28 (9.2%)	45 (20.7%)	< .001
I don’t seek care	79 (15.1%)	16 (5.2%)	63 (29.0%)	< .001
Call 811	38 (7.3%)	24 (7.8%)	14 (6.5%)	.546
Telus Babylon	38 (7.3%)	16 (5.2%)	22 (10.1%)	.033
Urgent care clinic	24 (4.6%)	8 (2.6%)	16 (7.4%)	.010
Virtual clinic	19 (3.6%)	8 (2.6%)	11 (5.1%)	.139
Other (e.g., ‘Naturopath’, ‘Pharmacist’, ‘Fill in locum doctors’)	46 (8.8%)	13 (4.2%)	33 (15.2%)	< .001
Number of HCP Visits				< .001
0 times	61 (11.7%)	19 (6.2%)	42 (19.4%)	
1-2 times	174 (33.3%)	98 (32.0%)	76 (35.0%)	
3-4 times	130 (24.9%)	72 (23.5%)	58 (26.7%)	
4+ times	158 (30.2%)	117 (38.2%)	41 (18.9%)	
What kinds of care did you receive (select all that apply)?^§^				
Non urgent visit due to a health issue	365 (69.8)	236 (77.1%)	129 (59.4%)	< .001
Urgent care due to a trauma, injury, or other emergency care	149 (28.5%)	94 (30.7%)	55 (25.3%)	.180
Screening/Preventative care	226 (43.2%)	159 (52.0%)	67 (30.9%)	< .001
Other (e.g. ‘surgery’, ‘prenatal care’, ‘medication renewal’)	73 (14.0%)	43 (14.1%)	30 (13.8%)	.941
Physical health				.791
Excellent	70 (13.4%)	43 (14.1%)	27 (12.4%)	
Good	267 (51.1%)	157 (51.3%)	110 (50.7%)	
Fair	137 (26.2%)	76 (24.8%)	61 (28.1%)	
Poor	38 (7.3%)	22 (7.2%)	16 (7.4%)	
Very poor	11 (2.1%)	8 (2.6%)	3 (1.4%)	
Mental health				.913
Excellent	72 (13.8%)	42 (13.7%)	30 (13.8%)	
Good	245 (46.8%)	146 (47.7%)	99 (45.6%)	
Fair	151 (28.9%)	85 (28.7%)	66 (30.4%)	
Poor	45 (8.6%)	28 (9.2%)	17 (7.8%)	
Very poor	10 (1.9%)	5 (1.6%)	5 (2.3%)	

^†^ n (%).

^‡^ Pearson’s Chi-squared test.

^§^ Percentages total more than 100, because participants could select all that apply.

HCP = health care provider.

#### Overall health and mental health

One overall physical and one mental health question was rated on a scale from 1 (very poor) to 5 (excellent). Single-item measures of health/mental health are widely used (e.g., Ware *et al.*, 1996) and have demonstrated reliability/validity (Ahmad *et al.*, 2014).

#### Thoughts and experiences with provider shortages

Participants were asked the following open-ended question: ‘What are your thoughts or experiences with the doctor shortage in BC?’.

### Analysis

Open-ended responses underwent iterative inductive thematic analysis following the approach outlined by Richards and Morse (2012). Text data extracted from survey responses were entered into an Excel spreadsheet that was organized according to attachment status. Open coding was performed for units of meaning (e.g., words, phrases, or paragraphs) for attached and unattached patients separately, and common meaning units were clustered into categories to construct an initial coding schema (KLR, CLS). Categories were similar across attached and unattached patients with variation across the two groups. Since there were more similarities than differences between attached and unattached patient experiences, the cross-group categories were merged, and categories abstracted to construct themes to reflect both groups. It was evident from the open-ended responses that the boundaries of attachment status were both fluid and blurry.

## Findings

During the approximately two-month recruitment period, 523 members from rural British Columbia communities participated in the survey, with 306 identified as attached and 217 as unattached. Participants had an average age of 50.69 years (Standard Deviation = 15.00) (range 19 to 86 years old). The majority were female (74.6%), Caucasian (90.1%), married or in a common-law relationship (68.3%), employed full-time (58.9%), and had household incomes over $75 000 (48.9%).

Overall, there were similarities and differences between unattached and attached patients in health characteristics and care seeking (see Table 1). In total, 17% of unattached participants had been without a health care provider (HCP) for 6 months or less, 54% 6 months to 5 years, and 29% had been unattached for over five years. Similarly, 9% of attached patients had been with their provider 6 months or less, 46% for 6 months to 5 years, and 45% had been with their provider for over 5 years. Expectedly, unattached patients were less likely to seek care from a regular provider and more likely to use walk-in, ED, Telus Babylon, urgent care, or not seek care at all compared to attached participants, with no differences in use of virtual clinics or 811. In terms of health characteristics, unattached patients had fewer HCP visits overall, received care for non-urgent and preventive reasons less often compared to attached patients, but these groups did not differ in self-reported physical or mental health (See Table 1).

### Thoughts and experiences with provider shortages

Of the 523 participants, 460 (88.0%) provided a response to the open-ended doctor shortage question; this included 191 (88.4%) of unattached patients providing responses, and 269 (87.6%) of attached patients providing responses. Both attached and unattached patients overwhelmingly described the healthcare system as ‘*broken’* and ‘*in crisis’*, even ‘*beyond a crisis*’. Three themes predominated patient responses: i) the ubiquity of the doctor shortage; ii) precariousness of attachment; iii) reflections on solutions. These themes are elaborated with participant quotes and excerpts (included in Supplemental File 1).

### Theme 1: the ubiquity of the doctor shortage

Attached and unattached patients alike acknowledged the presence of the doctor shortage. The majority described it as ubiquitous and pervasive, experienced at the national, community, family, and individual levels. Numerous participants had extreme, superlative descriptors to express the magnitude of the problem. One 58-year-old attached patient described, ‘*It is a disaster and only getting worse*’. A 73-year-old attached patient described it as, ‘*the most serious problem [facing] the country and our community*,’ [U187] and a 49-year-old unattached (5-10 years) female asserted, ‘*It is killing us and costing the system a lot of money on the way*’ (U2).

Attached and unattached participants voiced it as a pervasive Canadian problem, negatively comparing their care to other countries, groups, populations, or time periods. Common across participants’ responses was a comparison of their current care with third-world countries where they had received better care. They described medical care in Canada as ‘*disgraceful*’ ‘*unacceptable*’, ‘*ridiculous*’, ‘*archaic*’, ‘*criminal*’, ‘*worse than the care inmates receive*’, and ‘*Health services in this province (of British Columbia) were 1000% better in the 80s and 90s*’. [62-yr female; A768]

#### Ubiquity of the doctor shortages at a personal level

The majority of participants experienced the physician shortage up close and personal as they lived and heard about it daily and experienced it firsthand in their own lives or vicariously through family, friends, their rural communities, and across the nation. Participants varied in the extent to which they were touched by the doctor shortage. A minority, all of whom were unattached, described being distant from it, or that it was ‘*not a big deal*’ because they were ‘*in good health*’ or ‘*rarely go to the doctor anyway*’. Only one patient directly disagreed that there was a doctor shortage; a 44-year-old male described, ‘*It’s a sham. Doctors are there. You just have to look, make an appointment, or get referrals*’ [U66]. Paradoxically, this participant also reported not having a local, regular HCP for over 10 years.

Both attached and unattached participants described the negative impacts of the provider shortage on people’s health generally, and their own and their family’s health, specifically. A 37-year-old attached female participant highlighted generic impacts, ‘*It is terrible and is only leading to more advanced disease that is more costly to treat. There is a negative impact on quality of life for citizens*’. [A257]. Others described experiencing serious health risks from lack of provider oversight and timely access to emerging acute and ongoing chronic health problems. An unattached 61-year-old female [U78] relayed, ‘*My appendix ruptured last year and went septic because I had no doctor to examine me*’. A recently unattached (1-6 months) 54-year-old female expressed concerns about the negative impacts on chronic disease self-management, ‘*I have chronic issues which have been discussed and medication prescribed with my family doctor, who has moved away, and 3 locums. There has been no continuity and the medications are causing a lot of problems*’ [U791].

#### Ubiquity of doctor shortages at a collective rural community level

For rural participants, the provider shortage was not only an intensely individual issue but a collective problem that permeated the entire rural community. It touched whole rural communities directly through unattachment from the loss of the community’s doctor workforce. As an unattached 43-year-old female noted, ‘*Everyone we know who still had a family doctor in [northern rural community] has or is losing theirs imminently, including us… It’s stressful to lose (a) family doctor when we are due for a baby in 2 months*’. [U104]

The doctor shortage affected rural attached and unattached participants indirectly due to limited access and availability to ED, urgent care, and walk-in services regardless of attachment status. However, unattached compared to attached participants used all of these services to a much greater extent (see Table 1). A 43-year-old female attached patient of 5-10 years described the direct and indirect impacts of the shortage on BC residents as,

*‘It is a serious, urgent issue affecting all BC residents. ER’s and urgent care centres are filled with people who could get their care from a GP if they had one or didn’t need to wait weeks to see the one they have. Small communities like mine only have an ER, no walk-in clinic and a shortage of doctors so patients wait hours in the ER for in office level of urgency illnesses/issues’.* [A541]

So widespread was the use of ED, urgent care, and walk-in services that it had almost become a normal trend for rural participants. A 30-year-old recently unattached female described this normalization in their previous rural community, ‘*Something needs to be done to fix the shortage. I went years without a doctor and lived in a community where people regularly used the ER for routine care because there were no other options*’. [U128]

### Theme 2: precariousness of attachment status

Evident across both attached and unattached participants, was the overarching theme ‘precarious attachment’. Precarious attachment described participants emotionally charged experiences of uncertainty about their attachment status and reflected the most divergence between attached and unattached patients. The nature of this fluidity varied according to attachment status. For unattached participants it related to the uncertainty of if, and when, they would become attached while for attached participants it was the unsettledness of if, and when, they might become unattached.

#### Unattachment: abandoned, waiting, and trying to manage

For unattached patients the precariousness of attachment arose from the circumstances surrounding their unattachment and how they sought to manage it, the indefinite wait for attachment, and their health needs. Circumstances for unattachment varied. Some unattached patients had been given advanced notice without certainty about whether their Dr. would be replaced or the future of their attachment. Others described the sudden and unexpected loss of their doctors that in some cases was without notification, as a 32-year-old unattached (2-5 years ago) female shared, ‘*My doctor left, and the clinic dropped me as a patient without informing me. I called for an appointment and was informed I was no longer a patient. I have been unable to find another family doctor*’. [U141] Some patients moved or relocated only to find no available Drs in their new community, ‘*Couldn’t believe when I moved here that there were no doctors available (2-year waitlist to see a family doctor), not even a walk-in clinic. Didn’t even think about that when I moved here, assuming I would be able to find somewhere to go*’. [U40]

Unattached participants’ experiences of precarious attachment were most evident in the uncertain holding period of waiting for attachment. Several unattached patients (U61; U72; U163) talked about being on wait lists for protracted periods of time (> 10 years) since moving to the province. Others were unable to get on a waitlist, such as a 25-year-old unattached male, ‘*My partner and I can’t even get on a waitlist for a doctor in our area*’. [U163]

Unattached participants and those attached but who were previously unattached described the heightened impact of this precariousness if they had chronic disease management needs, were getting older and anticipated greater health needs, or were dealing with concerning health issues (e.g., precancerous cervical cancer). As one patient with chronic health concerns expressed,

*‘I’m shocked and angry that there is no access to a GP to work with me on my health and well-being at this stage of my life. There is no ability to get referrals to local BC specialists for specific care’.* [U183]

Surveillance and preventive care needs for some patients went unmet. As one patient noted,

*‘I was a patient of the clinic since 1984 and I feel like I’ve been abandoned. I have heart medications I take regularly which I can get renewed at a walk-in clinic, but they don’t monitor my lipid level or any ongoing preventative care. I worry that my cholesterol level or blood pressure will increase and no one will be monitoring’.* [U122]

Another younger patient echoed this concern,

*‘I’ve never had a regular GP in BC, and I’ve lived here for 12 years. I’m concerned about growing old here and inevitably getting more disabled--what will I do for preventative or ongoing healthcare? I deal with a lot of anxiety about this’.* [U169]

#### Attachment: still fraught with challenges and uncertainty

Though many attached patients were grateful to have a healthcare provider, the precariousness of attachment for patients of all ages stemmed from several sources: their experiences of how ‘*awful*’ and ‘*very hard*’ it had been to get a primary care provider; the loss of GPs (not their own) within the clinics they accessed or pending loss of their own GP as ‘*very concerning*’; and their general worries about their GPs retiring and uncertainty about their replacement. As one young patient shared, ‘*Getting my doctor was very hard, and if I were to lose her I know it would be impossible to find another doctor in the area…I greatly fear losing my family doctor and having a medical emergency*’. [A815] An attached 61-year-old voiced, ‘*I find that the constant anxiety of never knowing if a local doctor will be available and no specialists for any reason is extremely difficult*’. [A260].

#### Actions participants took in light of the uncertainty

The doctor shortage and the precariousness of attachment spawned participant caution in taking attachment for granted and consciously strategized ways to manage it including maintaining distant attachment and taking control of meeting health needs. Seventeen percent of unattached participants remained attached with their former regular care provider, travelling distances ranging from 46 to 4000 km and/or engaging virtually to receive care. According to a 63-year-old unattached (6 mo-2 years) female who had relocated and continued to travel 860 km, ‘*I’ve heard that it’s best to keep your existing physician if you move and rely on virtual contact with them (or go see them) as there is a severe lack in rural areas*’. This continuing but distant care was at great cost to participants who explained that they had ‘*pleaded with former doctor to conduct virtual appointment*’ and ‘*have not had a physical in years*’.

Participants also expressed the doctor shortage as an impetus for being more proactive in and taking control of their health needs. This included greater use of pharmacists and keeping track of their health records.

#### Attachment: should not be up to luck

Whether attached or previously unattached and now attached, the precariousness of attachment led participants to consider attachment as a matter of luck or good fortune. Even attached participants, some who had made great efforts to secure a provider, considered their attachment to be a stroke of luck. For some, attachments happened seamlessly because of an adequate GP supply in their rural community or because they were reassigned to another GP in the practice/clinic or a replacement for their GP was found.

Those who had been previously attached and now found themselves unattached reflected on how lucky they had been to have had regular providers for decades. A 40-year-old female recently unattached (within last 6 months) because of a move reflected this, ‘*I am realizing just how lucky and privileged my family has been with a family doctor for the past 20 years*’. [U178] However, a 39-year-old attached female (A930), who personally described her own luck in having a GP also rebutted that it shouldn’t be up to luck, and found the situation ‘*disheartening*’. ‘*I’m lucky to have a doctor, but this shouldn’t be the case that I’m ‘lucky*’.

### Theme 3: reflections on solutions

The overwhelming and passionate responses of participants to the ubiquity of the doctor shortage and their precarious attachment compelled a range of multi-faceted solutions to address the problem. Although responses varied, several participants elaborated thoughtful solutions that reflected their understanding of the complexity of the problem and the need for a multi-layered collective approach to address it. Whether attached or unattached, participants clearly wanted proactive solutions to the problem. Solutions included system, individual, and professional/regulatory solutions with the majority aimed at the system. System-level solutions called for government funding and support for physicians/healthcare professional compensation packages, including incentives advancing alternative reimbursement models. Medical school seat increases and policy development (e.g., rural retention) were other solutions. For example, student ‘*loan relief*’ for providers willing to ‘*stay in the smaller towns*’. As a 50-year-old attached male explained, “*A scale system of pay that rewards those willing to serve in much harsher and harder environments is not only fair but desperately needed* [A555]. A number of solutions advocated for ‘*system overhaul*’ and alternative healthcare system and primary care models of care, such as changing the culture to upstream prevention and wellness versus illness care and triaging pathways of care where ‘*doctors only need be closely involved with those who have life-threatening or altering diseases*’ [U843] Similarly, several solutions offered options for multi-professional team-based, holistic approaches that acknowledged the social determinants of health while removing the burden from a single provider. Likewise, individual-level solutions called for patient empowerment and support for ‘*home remedies/care*’ and ‘*the ability to handle small health concerns at home*’ [U766].

Professional/regulatory-level solutions were aimed at changing scopes of practice to expand the roles of nurses, nurse practitioners and allied health professionals in rural communities and streamlining foreign physician provincial licensing. A 57-year-old female described both the levelling of care and expanded nurse scope of practice,

*I think that levels of care (running on a scale of 1-10; 1-5 being basic check-ins, chronic health prescription renewal, and routine checks can be done by nursing staff… where more serious and urgent care for new illnesses 6-10 done by doctors) can be shifted which might lighten the responsibility to doctors. Also allow nurse practitioners to carry more of the load than doctors in rural areas. We have a couple of excellent ones.* [A275]

## Discussion

The purpose of this study was to investigate healthcare experiences of attached and unattached rural patients in the context of doctor shortages. Despite similar self-reported ratings of overall health, unattached patients received care less frequently compared to their attached counterparts, including less frequent non-urgent and preventive care. Unattached patients also demonstrated a higher propensity to utilize use walk-in, emergency departments, and urgent care facilities. While telehealth services (e.g., 811, Telus Babylon) were available to all British Columbians, smaller percentages of attached and unattached participants utilized such services. This set of findings suggests that these services do not fully close the gap created by shortages of primary care providers, forcing patients to seek more expensive urgent or ED care to have their healthcare needs met. In fact, Canadians appear to be the among the heaviest users of emergency departments; in a 2020 Commonwealth survey, 39% said their last ED visit was for a condition that could have been treated in primary care, if their regular providers had been available (Schneider *et al.*, 2021).

Most concerning in our study was the substantial percentage of unattached patients (29%) who reported not seeking care at all, mirroring findings by Marshall *et al.* (2022) in Nova Scotia, Canada, where unattached patients described ‘giving up’ on finding a healthcare provider and not seeking care (Marshall *et al.*, 2022). A scoping review of Canadians’ perspectives on the healthcare system found that, regardless of health stage/status, Canadians desired personalized, coordinated, and continuous care with regular contact with the same team of healthcare providers that was timely and minimized wait times (Peckham *et al.*, 2020).

Despite minimization of the doctor shortage by a handful of unattached, self-described healthy participants, the vast majority of respondents experienced the shortage as ubiquitous. Findings from this study are unique in uncovering the widespread and multi-level direct and indirect impacts of the doctor shortage, reflecting this as a persistent, dangerous problem that has worsened in Canada over time (Richardson and Hussain, 2022). Both attached and unattached participants experienced this system-level crisis and expressed a lack of confidence/uncertainty in their access to primary care – the cornerstone of Canada’s healthcare system which is intended to provide coordinated, continuous, high-quality care with referral to specialized services when required (Government of Canada, 2023a). This is especially problematic for those in rural communities where there are fewer healthcare options such as walk-in and urgent care facilities (Haggerty *et al.*, 2007). Attached and unattached response similarities reflect the impact of provider shortages on entire rural communities, as the services available are stretched to serve the residents.

Participants also described the nature of their care as precarious, expressing reluctance and embarrassment in using emergency services for non-urgent problems. Normalization and acceptance of services not intended for primary care reinforces operational inefficiency and wait times and delays and creates discontinuity and fragmentation of primary care and is the antithesis to better patient outcomes (Sandvik *et al.*, 2022; Jain, 2023). BC Medical Student recommendations included bolstering primary care as one avenue for reducing pressure on emergency departments, recognizing that for many patients, emergency departments are the only available healthcare option, so it’s not about inappropriate use, but access (UBC Medical students for the Provincial Advocacy Committee, 2024). Our findings point not only to the complexity of the doctor shortage but also to attachment status. It became clear that attachment was not a bipolar phenomenon, but that it occurred across a continuum that was highly fluid. There were participants who were unattached in their rural community who were receiving temporary care from a provider at a distance and there were participants who were technically attached but who expected to lose their provider imminently (e.g., to retirement) or received very irregular care, due to challenges with securing appointments and timely care. Indeed, according to an 11-country survey, less than half of Canadians are able to see a primary care provider within a day when they are sick (The Commonwealth Fund, 2020). Canada’s healthcare expenditures per person are amongst the highest Internationally (Canadian Institute for Health Information, 2023), yet, less of the total health budget (5.3%) in Canada is spent on primary care compared to other OECD countries (average 8.1%) (Shahaed *et al.*, 2023). The provider shortages have cyclical impacts for both attached and unattached patients alike, leading to costly system breakdown.

One short-term strategy used by our survey respondents was maintaining distant attachment with former providers. Since the COVID-19 pandemic, telehealth has taken a more prominent role in health care delivery, however, many clinicians are reverting to more in-person visits and some provinces are discontinuing temporary measures that paid for this type of care (Mehrotra *et al.*, 2020; Canadian Healthcare Technology, 2022). In addition, although authors of a systematic review found some benefits to using telehealth in primary care across all visit types, 40% of studies also reported drawbacks of telehealth including those involving new patients and for post-discharge follow-up (Ward *et al.*, 2022). Participants also reported finding ways to be more proactive in taking control of their health needs. With expansion of pharmacist roles in British Columbia and the adoption of virtual and hybrid clinics (e.g., FNHA Virtual Doctor of the day) and other innovations in care delivery, options for rural patients to receive needed care are improving (BC Pharmacy Association, 2023; First Nations Health Authority, 2024). Additionally, in BC forthcoming Registered Nurses may prescribe medications within their certified and scope of practice (BC College of Nurses and Midwives, 2024) and Real-Time Virtual Support provide clinical peer support (Rural Coordination Centre of BC, 2024). Further, efforts to provide patient navigation (Peart *et al.*, 2018) and self-management education and digital tools beyond specific disease conditions (McGowan, 2012) could also be enhanced as patients appear more motivated to have an active role in their health and health care.

Rural participants were not satisfied with merely reacting to the question inviting them to share their thoughts and experiences of the doctor shortage but offered numerous proactive solutions that reflected their investment in addressing the problem. Their solutions, related to compensation and financial support (e.g., incentives), reduction of providers’ administrative burden, facilitation of international medical graduate licensing, and expanding medical school seats, align with other pan-Canadian evidence (Li *et al.*, 2023). In contrast, Canadian Physicians and policy makers felt that attachment incentives could not overcome systemic challenges, as they did not address underlying provider shortages (Marshall *et al.*, 2023). Despite being one of the most populated provinces in Canada, BC has only one medical school, offering the least number of medical school seats (5) per 100 000 residents (Li *et al.*, 2023). Yet, training more providers is not the only solution to shortages, and our participants also recommended system reform. Others have also recommended approaches to support team-based care, sharing workload and administrative tasks, using virtual care, policy interventions, and neighbourhood-based care where akin to the educational system, families moving into a new location are guaranteed a spot in the local primary care practice (Kiran, 2022; Flood *et al.*, 2023; Nabieva *et al.*, 2023). A number of participants’ solutions were geared specifically to the rural context such as payment scales that recognizes the challenge of this setting or policy specific to rural retention. It is important that solutions be geared to the needs of rural communities and rural patients, who are immersed in the doctor shortage, and include them in addressing the problem. Although not a participant recommendation, other findings urge mechanisms for gradual retirement, succession planning, and public participation in public pension plans as potential solutions in addressing rural provider shortages (Hedden *et al.*, 2017). The findings presented here can be a springboard for more in-depth inclusion of rural voices in future work.

### Strengths and limitations

Despite the constraint imposed by the use of open-ended text limiting the opportunity to probe more deeply into participants’ experiences, this study was able to capture a comprehensive cross-section of rural participants from diverse rural geographical locations in the province. This inclusivity provided multiple and diverse perspectives that surfaced a more nuanced look at rural participants and the impact of the doctor shortage and the precariousness and the highly fluid nature of attachment that might not have been uncovered in a narrower sample of participants. Yet despite the diversity of experience and attachment status there was still a convergence of themes. Nevertheless, the online nature of the survey and convenience sample were limitations of the study. The sample being largely Caucasian, with 60% working full-time, and 50% with incomes over $75 000 limits generalization to other groups; in particular, inequities related to attachment and access are likely to be greater among those often marginalized in society and by health systems. In addition to primary care attachment, future work might include questions about availability of other health professionals (e.g., rehabilitation, social work, home care, and pharmacy) to better contextualize participant’s healthcare options.

### Conclusion

Despite similar overall health, unattached patients received care less frequently overall and were more likely to use walk-in, ED, and urgent care compared to attached patients. Yet, rural attached and unattached patients alike experienced provider shortages as ubiquitous, attachment as precarious, and expressed similar thoughts on solutions. Greater investment in primary care coupled with thoughtful policy response is urgently needed and wanted by rural British Columbians.

## Supporting information

Rush et al. supplementary material Rush et al. supplementary material

## Supplementary material

The supplementary material for this article can be found at https://doi.org/10.1017/S1463423624000677
