# Supplementary material for: The healthcare experiences of rural-living Canadians with and without a primary care provider: a qualitative analysis of open-ended cross-sectional survey responses
Source: Prim Health Care Res Dev. 2025 Jan 6;26:e1. doi: 10.1017/S1463423624000677 (PMC11735112; doi:10.1017/S1463423624000677)
Supplement: Rush et al. supplementary material [file S1463423624000677sup001.docx]

**Supplemental File 1. Themes and Additional Representative Quotes Regarding Thoughts or Experiences with the British Columbia Doctor Shortage**

| **Themes and subthemes, with representative quotes** |
| --- |
| **THEME 1:**  **Ubiquity of Doctor Shortage**  Definition: Pervasiveness of the doctor shortage, being constantly encountered and widespread and impacting both personal and community health.   - “I hear about Doctor shortages every day and it seems pretty bad - across the country.” [A330] - “A fact of life everywhere you go.” [A344]   **SUBTHEMES:**  ***Ubiquity of Dr shortages at a personal level***   - “It is what it is. I rarely go to the doctor anyways” [U69] - “My adult child, with Type 1 Diabetes cannot get a family Dr. which is worrisome. People's health suffers” [U85] - “People will have complications in their health which could have been prevented with regular doctor care, diagnostics, physical therapy. Complications and comprised health due to lack of access to specialists (no DR referral available)” [U726] - “I have full time health care needs with a part time doctor who does not have anyone to cover for when she is [not working]*.* There is no chance to get a different doctor as there are too many people without one….It prevents people from getting the care they need.” [A217]   ***Ubiquity of Dr shortages at a collective rural community level***   - “Walk in clinics are full as soon as they open and there are no physicians accepting new patients” [U4] - “It negatively affects rural communities. Whether you have an appointment to see the doctor that is cancelled due to emergencies or you’re relying on an emergency room being open - it is never certain your care will be timely.” [U146] - “I find it frustrating that patients need to utilize and take up emergency departments time for minor issues just because they do not have a doctor. It’s almost embarrassing to go to emerg for a prescription refill but we are left with no other choice.” [U86] - “Doctor shortage has seriously impacted our small town!!!! At one point we were down to one doctor. We have had our emergency department at our hospital closed on weekends multiple times since May and it is closed again this weekend!!” [A493] |
| **THEME 2:**  **Precariousness or Fluidity of Attachment Status**  Definition: Attachment status as fluid and ever-changing state, not fixed, but constantly in flux   - “I am losing my regular doctor and have to see a new one so I do not know how that is going to go. Hope for the best.” [A323] - “I would like to move but the doctor shortage is stopping me as I have one now and might not if I move.” [A71]   **SUBTHEMES:**  ***Unattachment: Abandoned, waiting, and trying to manage***   - “I just hope that I stay healthy. Going to the local health urgent care is traumatic especially as it is crowded and with few COVID restrictions in place…One doctor took me on as a new patient and then retired a month later.” [U540] - “I’ve been on the waiting list at my local clinic for 14 years. We are lucky to have a doctor and nurse practitioner here but demand vastly exceeds their availability and I’m worried what will happen when they retire.” [U588] - “For the first time in my life, I don't have a doctor, nor does my 78-year-old father. It feels very dangerous, as medical conditions we'd normally feel comfortable having looked at, now we put off because it's not an emergency, but it's still a health concern, but we have no one to take it to.”[U24]   ***Attachment: Still fraught with uncertainty***   - “I was without a doctor for a year, my husband currently does not have a family doctor, and as we age we will need access to healthcare.” [A554] - “I'm concerned that when my PCP retires, there may not be anyone to replace them. Our health center is on reduced hours (24hrs down to 12 hours as day). Loss of NP has affected my Mom's access to some degree as well, which affects me.”[A889] - “I was very happy to have an excellent family doctor, but have learned that he will be leaving our community in December. I am now very worried about whether or not he will be replaced and by whom” [A723] - “I still have a family doctor but I’m concerned about the near future because 3 of 6 doctors in our [rural community name] clinic are within 3 years of retirement.” [A101]   ***Managing uncertainty: Maintaining distant attachment*** ***and taking control of health needs***   - “I have to see my GP in Victoria, which usually means staying overnight, because the GP in [rural community] is not taking new patients. As a rural resident, I can't even see our health clinic for things like an infected bee sting or a sliver of glass in my foot because I am attached to a GP in the city.” [U717] - “Our doc in [former community] says he will keep us as long as he is able to have phone appointments. I feel so anxious that he will not be able to talk on the phone with us.” [U196] - “I am trying to take control of my health … I need to rebuild that relationship with a new provider, if a new provider is hired.” [A723] - “Since leaving Vancouver, our family has been completely on our own cobbling together care by asking pharmacists, keeping our own records for screenings (such as Pap) and then going door to door begging a physician for the screening, and hoping we're not missing something.” [A456] - “I will seek healthcare when I think I need it. I will just do the best I can for myself and forego professional directed preventative care until I can't avoid it.” [U454]   ***A matter of luck***   - “I have been lucky in that I found the First Nations Doctor of the Day program. It was a lucky break that I found it and maybe a bit of elbow grease on my part to pursue it.” [U199] - “We were lucky to get a family doctor after relocating to bc in 2020. However they are not a good fit and we are aware there are zero other options in our area. Doctors retire and/or burn out, and for some reason they do not want to move here.” [A902] - “I wish to see more drs so that everyone can have a family provider, I am one of the lucky ones who has one, but know many who don’t and makes it difficult to get proper care” [A920] |
| **THEME 3:**  **Reflecting on Solutions**  Definition: Solutions or recommendations to reduce the Dr shortage or the impacts of the shortage   - “Creating seats for nurses and doctors isn't going to solve the rural healthcare crisis if none of the seats are being filled with rural students who want to live rurally. A scale system of pay that rewards those willing to serve in much harsher and harder environments is not only fair but desperately needed. So is housing.” [A555] - “I don't have health problems or prescriptions, so I don't need a personal doctor. I think it's impossible for everyone to have a personal doctor who will treat all their medical/ psychomedical problems. I think a standard system of care through telephone advice, well-managed clinics, referrals, etc., would be fine for those without serious illness. Doctors only need be closely involved with those who have life-threatening or altering diseases.” [U843] - “I would like to see more general health care classes available to help with home remedies/care. Our system is overloaded because the ability to handle small health concerns at home has been taken out of the general populations hands. More education for self-care!” [U766] - “We do need to think more about addressing root causes of illness and not just treating symptoms; this places a lot of pressure on one provider to solve all patient issues, many that are often related to the social determinants of health. Having social worker, physiotherapy, counselors, social service organizations, and doctors more integrated to address holistic needs related to health and wellness. Fee for service doesn’t produce quality care, and if it does, it’s on the backs of those providers.” [A465] - “Shift in culture within primary care - willingness to share care across providers esp FPs…Expansion beyond ‘health’ to recognize that wellness and esp prevention are held as much in the community as in primary care - and social agencies are better at it. The silo of ‘health’ is a barrier to whole person preventative care” [U928] - “Maybe set up an incentive plan, that you stay in the smaller towns and your student loans are covered a % per year you stay” [U182]. - “The system needs to be completely overhauled so that doctors can be freed up to do what they do best and what they were trained for... being a doctor and not having to run clinics. Doctors need to be paid more and there needs to be more doctors so there is less of a burden on the existing ones. Doctors need more support - they should have a team including nurse practitioners, mental health specialists and other specialists. Doctors should have medical assistants to help with all the administrative tasks. They have so much paperwork which adds significantly more to the demands put upon them then simply the time spent seeing patients.” [A718] |
